# Mechanisms of Attenuation by Genetic Recoding of Viruses

**DOI:** 10.1128/mBio.02238-20

**Published:** 2021-01-05

**Authors:** Daniel Gonçalves-Carneiro, Paul D. Bieniasz

**Affiliations:** a Laboratory of Retrovirology, The Rockefeller University, New York, New York, USA; b Howard Hughes Medical Institute, The Rockefeller University, New York, New York, USA; Academic Medical Center of the University of Amsterdam; Albert Einstein College of Medicine

**Keywords:** codon, RNA, virus, translation

## Abstract

The development of safe and effective vaccines against viruses is central to disease control. With advancements in DNA synthesis technology, the production of synthetic viral genomes has fueled many research efforts that aim to generate attenuated viruses by introducing synonymous mutations. Elucidation of the mechanisms underlying virus attenuation through synonymous mutagenesis is revealing interesting new biology that can be exploited for vaccine development. Here, we review recent advancements in this field of synthetic virology and focus on the molecular mechanisms of attenuation by genetic recoding of viruses. We highlight the action of the zinc finger antiviral protein (ZAP) and RNase L, two proteins involved in the inhibition of viruses enriched for CpG and UpA dinucleotides, that are often the products of virus recoding algorithms. Additionally, we discuss current challenges in the field as well as studies that may illuminate how other host functions, such as translation, are potentially involved in the attenuation of recoded viruses.

## INTRODUCTION

A core property of nucleic acids is the ability to store and communicate information encoded in the order of constituent nucleotides. Genes are composed of linear arrays of codons that are translated by ribosomes to produce polypeptides: a flow of information that is universal in both cells and viruses. Because a total of 61 codons can be translated into 20 amino acids (plus termination codons), redundancy in the genetic code allows organisms to develop and maintain coding biases. Coding biases occur when certain codons, codon pairs, or nucleotide combinations are enriched or depleted in protein-coding sequences. These biases can influence gene expression by altering either mRNA stability or translation efficiency. Consequently, viruses are subjected to host-imposed pressures on coding sequences, and some viruses appear to have nucleotide compositions that have been selected to optimize replication in particular hosts.

Recent advances in DNA synthesis have impacted research that aims to understand coding biases and how synonymous mutations might affect cellular and viral processes. Disrupting coding biases by large-scale genomic recoding of viruses can lead to substantial reductions in viral fitness. Thus, the recoding and synthesis of entire viral genomes have created a platform for the generation of live, attenuated vaccines, i.e., viruses that carry synonymous mutations and have suboptimal replication cycles both in cell culture and *in vivo*, as well as a platform to investigate the underlying molecular biology of viruses (1, 2).

The methods and approaches to virus recoding through codon or codon pair deoptimization approaches, manipulation of the dinucleotide content, and other genomic alterations of viral genomes have been extensively reviewed elsewhere (1, 2). Here, we review the biological mechanisms underlying the attenuation of recoded viruses. We highlight the activity of certain antiviral proteins and their links to the nucleotide composition of virus genomes. We also discuss how recoding approaches can impact translation efficiency and promote viral RNA degradation. Understanding the mechanisms underlying attenuation following large-scale genomic recoding of viruses should impact vaccine design and potentially other therapeutic approaches.

## CODING BIASES IN CELLULAR AND VIRAL GENOMES

The nucleotide sequence does not solely specify the order of the amino acids to be translated but also can impact translation efficiency (3), mRNA stability (4), and the subcellular localization of an mRNA molecule (5). A prominent example of nucleotide compositional bias is the extreme suppression in human genomes—and, more generally, genomes of vertebrates—of CpG dinucleotides (6). The tendency to avoid CpG dinucleotides is not observed in the majority of invertebrates, some clades of plants, and bacteria, whose DNA genomes exhibit higher CpG dinucleotide frequencies (7). On the other hand, UpA dinucleotides (or TpA in DNA) are underrepresented in virtually all living forms (8). Variation in the degrees to which these dinucleotides are suppressed within genomes is observed: in humans, CpG dinucleotides are suppressed in all genomic DNA, while UpA dinucleotides are mostly suppressed in cytoplasmic RNA sequences (8, 9).

Two hypotheses might be advanced to explain compositional biases. The first posits that dinucleotide and codon biases arise from underlying mutagenic sources and that synonymous mutations arise without affecting the fitness of the gene or the organism (10). An example of such a mutational process is that which gives rise to the CpG-suppressed state of human genomes. In DNA, cytosines spontaneously undergo oxidative deamination at a low rate. Ordinarily, cytosine deamination generates uracil, which is recognized by DNA repair mechanisms and corrected to the original G:C base pair. However, cytosines in a 5′-CpG-3′ context are often substrates of DNA methyltransferases (11). The product of this reaction is a 5-methyl-cytosine whose deamination generates thymine, generating a G:T rather than a G:U mismatch, which is less frequently “correctly” repaired to the original G:C base pair. This process appears to be responsible for the naturally occurring depletion of CpG suppression (12). In fact, the absence of DNA methyltransferases in certain species of invertebrates correlates with the higher CpG frequencies observed in their genomes (13, 14). An alternative hypothesis that might drive compositional bias argues that synonymous mutations influence the fitness of an organism and are therefore selected. Evidence in support of this hypothesis includes the correlation between preferred codons in highly expressed genes and the abundance of the cognate tRNA (15, 16), suggesting that selection for translation efficiency might impact nucleotide composition. In reality, both hypotheses likely contribute to the generation and maintenance of codon or dinucleotide biases in host genes.

Viruses that infect eukaryotes mimic, to some extent, the compositional biases of their hosts (17, 18). For example, viruses that replicate in mammals have lower CpG dinucleotide contents than viruses that replicate in insects (9). Correspondingly, mammalian genomes have fewer CpG dinucleotides than expected, but insect genomes do not. Additionally, viruses that infect plants are profoundly suppressed in UpA dinucleotides, mimicking the low level of TpAs present in plant genomes (19). Indeed, using machine learning approaches and large viral ecological data sets, one study suggested that virus reservoirs can be predicted based on the coding biases of both viral and host RNA sequences (20). Key evidence in support of the notion that viral mimicry of host nucleotide composition is a selected property is provided by a longitudinal study of H1N1 influenza A viral genome sequences (21). H1N1 influenza A virus (IAV) is believed to have been transmitted from birds to humans, giving rise to the infamous 1918 pandemic. Initially, this H1N1 virus had a relatively large number of CpG dinucleotides, but viruses subsequently isolated from humans over the ensuing century have progressively fewer CpGs in their genomes. This observation suggests that host-specific selective pressures drive compositional biases in viral genomes.

## APPROACHES TO VIRAL GENETIC RECODING

Generally, the main goal of genetic recoding of viruses is to identify intrinsic coding or compositional biases of viral genomes that facilitate replication and deliberately disrupt such biases. An effectively attenuated virus will ultimately have fitness that is reduced *in vivo* to the extent that it is apathogenic yet retain sufficient levels of replication to elicit a strong immune response. One of the advantages of using genomic recoding to generate attenuated viruses is the inherent stability of the introduced mutations, as the fitness deficits imposed by each nucleotide substitution are imperceptible, but their cumulative effect can be substantial. Conversely, live attenuated vaccines that carry a small number of amino acid substitutions might easily reacquire wild-type fitness following immunization. A well-known example is the live, attenuated poliovirus vaccine that encodes 3 amino acid substitutions that significantly reduce virus replication. However, reversion occurs *in vivo*, and neurovirulence is evident following transmission (22). Indeed, most current cases of poliovirus infection involve a reverted vaccine strain and not wild-type virus. Few studies examined the stability of recoded genomes. Several codon pair-deoptimized, temperature-sensitive respiratory syncytial virus (RSV) mutants were serially passaged under consecutively higher-temperature culture conditions; however, no virus adaptation or reversion was observed (23). Nonetheless, one mutant virus that contained a smaller number of mutations regained fitness, possibly by increasing the transcription of the recoded gene without losing any attenuating mutations. Another study showed that long-term passage of a codon-deoptimized poliovirus resulted in the progressive purging of introduced CpG dinucleotides from its genome (24). Thus, while a small number of introduced mutations may be insufficient to maintain virus attenuation, large-scale genetic recoding approaches of viruses are likely to generate stable attenuated viruses that may be used as vaccine candidates.

### Modulation of codon and dinucleotide frequencies.

**FIG 1:**
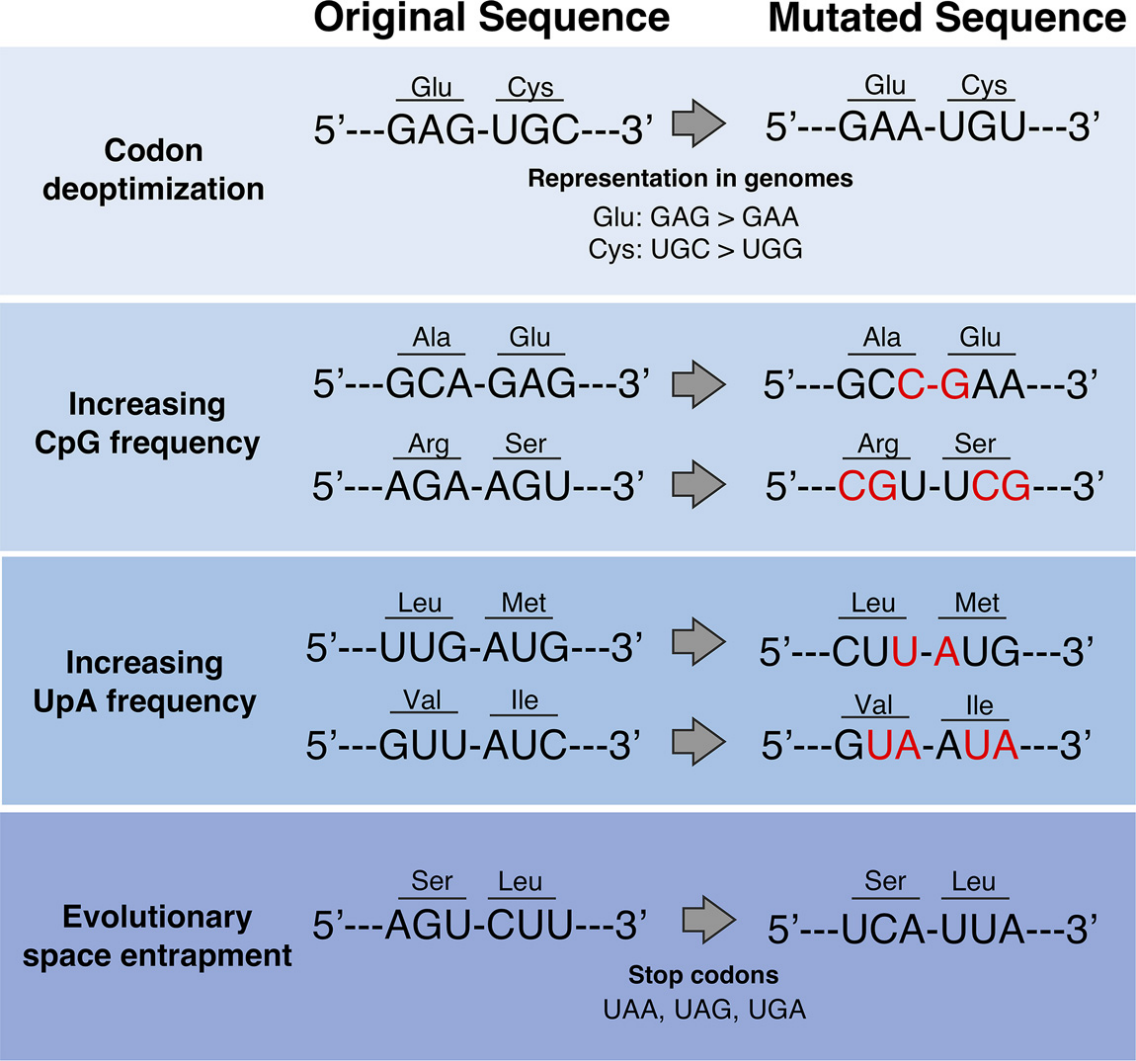
Codon substitution approaches used in genetic recoding of viruses. Several synonymous mutagenesis approaches may be applied to generate attenuated viruses. Codon deoptimization aims to replace highly frequent codons (such as GAG for glutamate and UGC for cysteine) with underrepresented codons (such as GAA and UGU, respectively). Codon substitution may also lead to increased CpG and/or UpA frequencies by introducing these dinucleotides either at the codon-codon boundary (e.g., replacing the GCA-GAG pair with GCG-GAG) or within a codon (e.g., AGA-to-CGU substitution). Replacing serine and leucine codons with “near-stop” codons (i.e., AGU and CUU to UCA and UUA) may also lead to viruses whose replication is aborted at unusually high frequencies through the frequent generation of mutants expressing truncated viral proteins.

Most previous studies have employed “codon pair deoptimization” as an approach to attenuate viruses. Codon pair deoptimization is achieved by replacing frequently observed codon pairs with infrequently used codon pairs (Fig. 1). This approach has shown to be effective in a wide range of viruses, including picornaviruses (25–27), flaviviruses (28), orthomyxoviruses (29), pneumoviruses (30), herpesviruses (31), arenaviruses (32), and lentiviruses (33). Most of these viruses are human pathogens, and codon pair deoptimization gives various levels of attenuation, as assessed by replication in human cells. Attenuation has been observed both *in vitro* and in animal model systems (30, 34). In some studies, recoded viruses that are transmitted by mosquitos were shown to be significantly attenuated in human cells but not insect cells (35). This finding suggested that codon pair deoptimization has host-specific effects and is not a universal mechanism of attenuation. Although codon pair deoptimization was initially and reasonably thought to exert its effects through decreased translation efficiency, codon pair deoptimization also perturbs dinucleotide composition. Indeed, codon pair deoptimization results in viruses with elevated levels of CpG and UpA dinucleotides, primarily by enriching these dinucleotides at codon boundaries (Fig. 1). Increasing the frequency of these dinucleotides in viral genomes has been shown to attenuate viruses in hosts as divergent as humans and plants (19, 36–39). Recoding methodologies that disrupt codon usage, not codon pairs, have also been successful in generating attenuated viruses. Such studies aimed to mimic the codon usage of avian influenza viruses in human IAV (40) or to mimic the less frequent codons in humans (41). However, in these cases, the investigators inadvertently increased the frequency of CpG and UpA dinucleotides. Increased levels of CpG/UpA dinucleotides have been found to be deleterious for virus replication in both coding and noncoding regions of RNA molecules (36, 42, 43). Further investigation is required to tease apart the effects of codon and codon pair deoptimization and increases of CpG/UpA dinucleotides on viral replication. A systematic approach that uses identical recoded sequences in either translated or untranslated regions of viruses might be useful to elucidate the direct role of dinucleotides in RNA versus modified codons in a sequence.

### Evolutionary space entrapment.

A consequence of genetically recoding viral genomes is the reframing of the “evolutionary space.” During the propagation of viral populations, nucleotide and protein sequences that contribute to high fitness are selected. An optimized nucleotide sequence results in a given set of protein variants that can be easily accessed by nucleotide substitutions (44). Recoding viral genomes may reframe the position of that viral sequence in evolutionary space, change the spectrum of protein variants that can be accessed by nucleotide substitutions, and sensitize a sequence to mutation. One approach to exploit this concept for the attenuation of viruses is the substitution of leucine and serine codons, the two most redundant codons in the genetic code, for “near-stop” codons, i.e., codons in which a single nucleotide substitution can generate a nonsense mutation (45) (Fig. 1). Indeed, coxsackie B3 viruses and IAVs that were recoded to contain numerous near-stop codons were shown to exhibit severe replication defects in the presence of mutagenic compounds. Analysis of infected cells revealed the presence of viral genomes with acquired stop codons. On the other hand, reframing of viral evolutionary space may also facilitate the acquisition of resistance to antiviral drugs. Indeed, a mutant of human immunodeficiency virus 1 (HIV-1) that carried several synonymous mutations in the HIV-1 protease-coding sequence (46) was found to more easily adapt to the presence of protease inhibitors than the parental, wild-type virus.

## CpG DINUCLEOTIDE-INDUCED ATTENUATION

**FIG 2:**
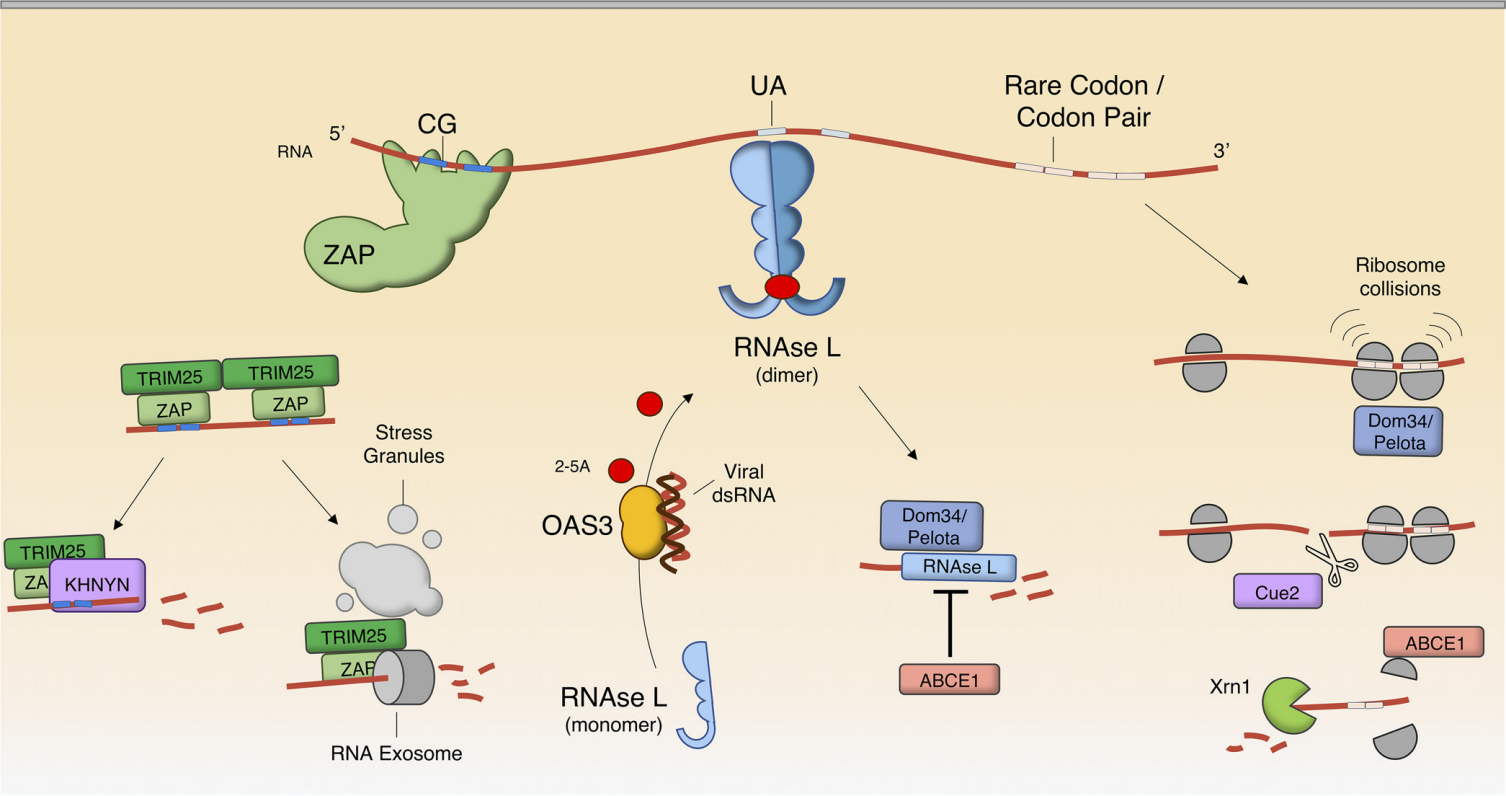
Molecular mechanisms that limit the replication of recoded viruses. Several elements present in viral RNA may be recognized and eliminated by various mechanisms. CpG-rich RNA is detected by ZAP, whose interaction with TRIM25 may facilitate the coalescence of other cellular proteins that determine its fate. Viral RNA that is recognized by ZAP can be degraded by the endonuclease KHNYN or relocalized to stress granules, where it may become a substrate of the RNA exosome. The presence of dsRNA, a frequent product of virus replication, is detected by OAS3, leading to the production of 2′-5′-oligoadenylate (2-5A) from ATP. The ankyrin repeats of RNase L interact with 2-5A, promoting the formation of a dimeric, active state of this protein. RNase L interacts with Dom34/Pelota and cleaves mRNA 3′ to UpA dinucleotides. This reaction is inhibited by ABCE1. The presence of rare codons or codon pairs may lead to slow ribosome translocation, causing ribosome collisions. Stalled ribosomes are sensed by Dom34/Pelota, recruiting Cue2/N4BP2 that cleaves the translating mRNA. Ribosome dissociation is promoted by ABCE1, while Xrn1 and other exonucleases degrade mRNA containing inhibitory codon pairs.

The introduction of CpG dinucleotides in the genomes of viruses, either deliberately or as a consequence of codon/codon pair deoptimization, frequently results in reduced viral fitness (37, 38, 43). The major mechanism by which CpG-enriched viruses are attenuated is through recognition by the zinc finger antiviral protein (ZAP) (Fig. 2). ZAP was initially identified as an inhibitor of Moloney murine leukemia virus (MLV) (47), and it has since been implicated in the inhibition of a broad range of viruses, including alphaviruses (48), filoviruses (49), flaviviruses (50), and hepadnaviruses (51). However, other viruses, such as yellow fever virus and vesicular stomatitis virus (VSV), were reported to be insensitive to ZAP (48). ZAP is composed of an N-terminal RNA-binding domain (comprised of four CCCH-type zinc fingers), two WWE domains, and a catalytically inactive poly(ADP-ribose) polymerase (PARP)-like domain. Early studies showed that the antiviral activity of ZAP was strictly dependent on its RNA-binding domain and suggested that ZAP recognized and bound to viral RNA (52, 53). Indeed, in some cases, the expression of the RNA-binding domain of ZAP alone was sufficient to inhibit virus replication (47). Early attempts to determine what RNA features or sequences were recognized by ZAP failed to identify any consensus sequence or structure (49, 53). Later, it became clear that CpG dinucleotide enrichment rendered HIV-1 sensitive to the expression of ZAP (43). Indeed, CpG-enriched HIV-1 mutants were found to be substantially attenuated in ZAP-expressing cells but replicated at near-wild-type levels when ZAP was absent. Similarly, CpG-enriched echovirus 7 mutants (42) were attenuated in a ZAP-dependent manner. Reanalysis of previously identified ZAP-responsive elements revealed that they, as well as viruses that were previously shown to be sensitive to ZAP, contained high frequencies of CpG dinucleotides (43). Using crosslinking immunoprecipitation coupled with RNA sequencing (CLIP-Seq) approaches, it was found that ZAP indeed specifically bound to CpG-rich RNA elements (Fig. 2). Moreover, structural analyses have recently demonstrated that ZAP selectively binds to CpG dinucleotides through residues located primarily in the second zinc finger: a hydrophobic pocket therein can accommodate only CpG dinucleotides (54, 55). Mutagenesis in and around the CpG-binding site in ZAP caused a loss of specific antiviral activity against CpG-enriched HIV-1 (54). A recent study reported that certain areas of HIV-1 genomes are more sensitive to the introduction of CpG dinucleotides, and, hence, to the activity of ZAP, than other regions (56). It is possible that sequence context and CpG distribution might affect sensitivity to ZAP, and further investigation is required to clarify this topic. Collectively, these data indicate that ZAP is responsible for the attenuation of CpG-enriched viruses, in at least some instances, and that ZAP recognition of CpG dinucleotides in viral genomes is essential for its antiviral activity.

ZAP is ubiquitous in human tissues, but its levels can also be increased upon viral infection or stimulation with type I interferons (IFNs) (57). IFN-mediated ZAP induction is dependent on IFN regulatory factor 3 (IRF3) and IRF3-binding sites in the *ZAP*/*ZC3HAV1* promoter region (58). The *ZC3HAV1* gene encodes multiple ZAP isoforms, most prominently the so-called long (ZAP-L) and short (ZAP-S) isoforms, which differ from each other in the presence or absence of a PARP-like domain. Two additional isoforms, the medium (ZAP-M) and extralong (ZAP-XL) isoforms, resulting from the extended readthrough of exon 4 are also expressed in human cells (59). All ZAP isoforms have antiviral activity, but their potency varies somewhat (59). While little is known about the mechanism underlying the differential potencies of the isoforms, differences in cellular localizations may contribute (59, 60). How the expression of each isoform is regulated is not completely understood, but two cellular mRNA antiterminator proteins (SCAF4 and SCAF8) are reported to modulate the expression of ZAP-L and ZAP-S (61). These proteins bind a polyadenylation signal located 5′ to the terminal exon of *ZC3HAV1*, preventing transcription termination by masking this site, thus favoring the expression of ZAP-L. When SCAF4 and SCAF8 are absent, the otherwise masked polyadenylation site is used, favoring ZAP-S expression. Plausibly, the expression of different ZAP isoforms in different tissues may affect virus replication and the *in vivo* biological impact of recoded viruses.

Early studies demonstrated that viral RNA levels were reduced in the presence of ZAP (47, 53, 62). This apparent destabilization of viral RNA was shown to be limited to the cytoplasm, where ZAP is present, and nuclear viral RNA was not affected (43, 47). Some studies have suggested that ZAP can also destabilize certain retrotransposons and CpG-rich cellular mRNAs (63, 64). The process by which ZAP induces cytoplasmic RNA depletion is thought to involve the recruitment of nucleases (Fig. 2). Some studies have suggested that the RNA-binding domain of ZAP binds components of the RNA exosome such as EXOSC4 and EXOSC5 (65) and that such proteins are important for anti-MLV and anti-Japanese encephalitis virus activity as well as ZAP-dependent depletion of *TRAILR4* mRNA (50, 63). ZAP has been found to colocalize with EXOSC5, recruiting both target RNA and exosome components to stress granules (64, 66, 67). However, other studies have reported that exosome component depletion did not appreciably rescue CpG-enriched HIV-1 replication (43). Moreover, pharmacological inhibition of the exosome only marginally increased hepatitis B virus RNA levels (51). Recently, a different putative endonuclease, KHNYN, was shown to bind ZAP and to be essential for ZAP-dependent inhibition of CpG-enriched HIV-1 replication (68). However, KHNYN knockout did not rescue Sindbis virus or MLV replication (68). One explanation for these discrepancies may be that ZAP recruits different RNases in different viral contexts (Fig. 2). Consistent with this idea, MLV and Sindbis virus infections lead to the formation of ZAP-positive stress granules (66, 69), while HIV-1 infection does not (70). Possibly, subcellular localization determines which RNases ZAP interacts with; perhaps, ZAP recruits exosome components to stress granules but binds KHNYN in the cytosol.

Another cofactor, TRIM25, an E3 ubiquitin and IFN-stimulated gene 15 (ISG15) ligase, was reported to be required for ZAP antiviral activity (71, 72) (Fig. 2). TRIM25 contains a RING domain, which mediates the transfer of ubiquitin to the target protein, and is known to ubiquitinate several substrates, most notably RIG-I and MDA5. The TRIM25 SPRY domain is known to interact with and provide specificity for E3 ubiquitin ligase substrates, such as the CARD domain of RIG-I (73). The SPRY domain was also shown to be important for ZAP binding and cofactor activity (71). While a few studies have demonstrated that certain lysine residues in ZAP are ubiquitinated by TRIM25 (71, 72, 74), ZAP ubiquitination does not seem to impact its antiviral activity (71, 72). Nevertheless, a truncated form of TRIM25 that lacks its RING domain or a catalytically inactive mutant (C50S,C53S) does not support ZAP antiviral activity. While these findings are consistent with the idea that the ubiquitin ligase activity of TRIM25 is important for antiviral effects, perturbing the RING domain, either by introducing mutations or by removing it entirely, also disrupts TRIM25 oligomerization. Homotypic interactions between the coiled-coil domains drive the formation of antiparallel TRIM25 dimers (75), while the interaction of two RING domains drives higher-order multimerization and is necessary for its ubiquitin ligase activity (76, 77). Additionally, two recent papers have reported that TRIM25 was also capable of binding RNA, despite disagreement over which TRIM25 domains mediated this interaction (74, 78). One report indicated that a patch of hydrophobic amino acids within the SPRY domain binds RNA and that RNA binding is essential for the *in vitro* ubiquitination of ZAP (74). In contrast, another report indicated that the SPRY domain alone cannot bind RNA *in vitro* and that seven lysine residues located between the coiled-coil domain and the SPRY domain drive RNA binding (78). Clearly, the ability of TRIM25 to bind RNA is likely to be important for its mechanistic role in the antiviral activity of ZAP. Given its propensity to form higher-order multimers, TRIM25 may function as a nucleation factor where several proteins, including ZAP and KHNYN, coalesce to degrade viral RNA. Evidently, further work is required to elucidate the role of TRIM25 in ZAP activity.

While the above-mentioned studies suggest a direct role for ZAP and associated proteins in the depletion of viral RNA from infected cells, an additional mode of action of ZAP might be through sensing and signaling. One study showed that ZAP overexpression in the presence of RIG-I agonists, or during IAV and Newcastle disease virus infections, caused increased production of IFN-α and IFN-β (79). ZAP-S, in particular, activated both the NF-κB and IRF3 transcriptional pathways in a manner that was dependent on RIG-I and MAVS (79). In contrast, ZAP-S was also reported to facilitate the resolution of innate immune responses (80). Specifically, ZAP-S was reported to limit the expression of certain IFN genes. This finding is concordant with previous results that indicated that Sindbis virus infection of ZAP-deficient mice led to high immune responses and survival rates compared to wild-type mice (81). Another study reported that CpG-enriched IAV had replication defects in mice, while host immune responses were substantially exacerbated compared to wild-type virus infection (37). Others have shown that treatment of primary human plasmacytoid dendritic cells with short CpG-rich RNAs increased the production of IFN-α (82). It is paradoxical that ZAP might both enhance an innate immune response and limit the duration of the expression of IFN genes, and in animal model systems, the level of viral replication is an obvious important confounding variable. Careful delineation of the spatial and temporal parameters with which these events take place may be key to understanding these putative roles of ZAP in the establishment and modulation of innate immune responses.

Together, these findings suggest that while ZAP has broad antiviral activity that is mobilized by CpG dinucleotides present in viral genomes, its mechanism of action may be complex. Fundamental to the understanding of ZAP activity is the elucidation of subcellular localization and its interaction partners. ZAP may restrict CpG-enriched virus replication by a variety of mechanisms, including RNA degradation, translation inhibition, and the induction of an innate immune response.

## UpA DINUCLEOTIDE-INDUCED ATTENUATION

The dinucleotide UpA is almost universally suppressed in all life forms and viruses. The addition of UpA dinucleotides to viral genomes, which are frequently present only at low levels, can lead to a loss of viral fitness for picornaviruses, flaviviruses, and potyviruses (35, 36, 38, 39). Curiously, UpA-enriched echovirus 7 mutants were found to be selectively attenuated in unmanipulated cells but replicated with near-wild-type kinetics in ZAP knockout cells (42). This finding is unexpected because CLIP-Seq (43) and crystallographic studies (54, 55) have indicated that ZAP binds selectively to CpG dinucleotides in viral RNA. Even though UpA dinucleotides are unusually abundant in HIV-1, no specific interaction was observed between ZAP and these dinucleotides (43). One possible resolution of this apparent contradiction may be that U- and A-rich RNA molecules are less likely to form secondary structures. Thus, increasing the UpA content of viruses may increase the exposure of CpG dinucleotides, facilitating recognition by ZAP. In all published structures of the N-terminal domain of ZAP, several hydrophobic pockets were predicted, but only one of them interacted with CpG dinucleotides (55, 83). Perhaps, in some circumstances, those regions might interact with UpA dinucleotides; however, no evidence of that possibility was found in infected cells (43). Further investigation is required to assess the precise role of ZAP in the inhibition of UpA-enriched viruses.

Knockout of RNase L or 2′-5′-oligoadenylate synthase 3 (OAS3) has been reported to alleviate, to some extent, the replication defect in certain compositionally altered viruses (42). The OAS3/RNase L system that responds directly to the presence of double-strand RNA (dsRNA) has been reported to inhibit the replication of several viruses, including encephalomyocarditis virus (EMCV) (84), coxsackievirus B4 (85), and Theiler’s virus (86). Viral dsRNA formed through replication complexes of RNA molecules with opposite polarity or through stem-containing secondary structures can be recognized by different OAS paralogs (OAS1, OAS2, and OAS3). OAS proteins catalyze the production of 2′-5′-oligoadenylate from ATP (87–89), which is recognized via nine N-terminal ankyrin repeats, present in RNase L. The 2′-5′-oligoadenylate–ankyrin repeat interaction leads to the formation of an active RNase L dimer (90–92). Notably, OAS3 seems to be a more potent activator of RNase L than OAS1 and OAS2 during virus infection (93–95).

The RNase activity of RNase L targets RNA immediately 3′ to UpA and UpU dinucleotides (96, 97) (Fig. 2). Indeed, hepatitis C virus (HCV) genomes with higher frequencies of UpA and/or UpU dinucleotides were found to be more sensitive to RNase L (98), and circulating HCV genotypes that are more resistant to the effects of IFN contain lower levels of these dinucleotides (99). RNase L was also reported to preferentially cleave at UpA or UpU dinucleotides in poliovirus and IAV RNAs (100, 101). Thus, viruses with fewer UpA or UpU dinucleotides appear more resistant to RNase L. However, UpU dinucleotides are not underrepresented in human genomes or viruses that infect humans. Possibly, the UpU motif targeted by RNase L may be part of a more complex sequence that determines substrate specificity. Indeed, while increasing the frequency of UpA dinucleotides in viral RNA is likely to lead to virus attenuation via the OAS3/RNase L pathway, further investigation is required to address whether this effect is recapitulated by the elevation of the UpU dinucleotide frequency.

A cofactor for RNase L, termed Pelota/Dom34, has recently been identified (102) via its apparent ability to target EMCV RNA for degradation and thereby inhibit replication. Pelota directly binds RNase L, and it is a homolog of the gene *Dom34* in Saccharomyces cerevisiae that is a component of the No-Go RNA decay pathway (103) (Fig. 2). In yeast and drosophila model systems, the no-go decay pathway is responsible for the degradation of mRNA with stalled or collided ribosomes that arise if translating mRNA contains rare codons, rare codon pairs, or stable secondary RNA structures (104–106). Additionally, an ATP-binding protein termed ABCE1 was shown to regulate the activity of RNase L. ABCE1 inhibits the antiviral activity of RNase L against EMCV (107) and was reported to be essential for infection by viruses that are naturally UpA rich (108, 109). Overall, the OAS3/RNase L/Pelota axis appears important for the attenuation of UpA-enriched viruses. Notably, however, viruses have evolved a plethora of mechanisms to counteract the activity of the OAS3/RNase L pathway (86), and this may explain why CpG suppression is more striking than UpA suppression in mammalian viruses (9).

Induction of the innate immune response upon the sensing of UpA-rich RNA may represent a further mechanism of UpA-induced viral attenuation. While OAS3 and RNase L are induced by IFN (110), they are constitutively present in most human tissues. Indeed, in experiments where fibroblasts were treated with 2′-5′-oligoadenylate or infected with Sendai virus, IFN-β production was dependent on RNase L expression (111). Moreover, short, UpA/UpU-containing RNAs, which are the products of the RNase L action on HCV genomes or host RNAs, activated RIG-I and MDA5-dependent signaling pathways (111, 112). Interestingly, while RNase L activation can cause translation arrest and global mRNA turnover, mRNAs encoding certain antiviral proteins, in particular *IFNB* and *IFNL* mRNAs, are resistant to this degradation (113, 114). Additionally, during IAV and VSV infections, the RNA products of RNase L were reported to activate the NLRP3 inflammasome in a MAVS-dependent but RIG-I/MDA5-independent manner, leading to the production of several cytokines, including interleukin-1β (IL-1β) (115). Together, these findings suggest that RNase L exerts antiviral activity both directly, by degrading UpA-rich RNA, and indirectly, by facilitating the establishment of an IFN-induced antiviral state.

## CODON- AND CODON PAIR-DERIVED ATTENUATION

Approaches that aim to disrupt inherent codon or codon pair biases frequently result in virus attenuation. However, because of the confounding effects of enriching CpG and UpA dinucleotides that accompany these manipulations, it is difficult to disambiguate the underlying mechanisms (38). Nevertheless, it is plausible that additional mechanisms that are not directly related to CpG or UpA content contribute to virus attenuation following genetic recoding. Notably, not only codon pair deoptimization but also codon pair optimization was reported to diminish RSV replication (116), and both types of perturbation resulted in a temperature-sensitive phenotype, with reduced replication at 37°C but not at 32°C (30). Codon optimization of the adenovirus fiber protein can also lead to virus attenuation (117), even though codon optimization resulted in increased protein expression.

The study of codon usage has greatly improved our understanding of relationships between mRNA stability and translation (118). Genes that contain frequently used codons were thought to be more highly expressed due to a higher abundance of cognate tRNAs, as predicted by the number of tRNA genes present in a genome (119). However, tRNA gene number does not accurately predict tRNA abundance (120). In a study that assessed the ability of all possible codon pairs to promote or repress the expression of a fluorescent reporter gene in yeast (121), 17 codon pairs were found to be associated with reduced reporter expression. Of these, 16 contained at least one CpG dinucleotide. In this study, translation was sensitive to the order of codons; i.e., reversal of the codon order improved translation. Interestingly, the majority of codon pairs contained a codon that relied on wobble decoding, and mRNAs containing inhibitory codon pairs had elevated ribosome occupancy. Crucially, the inhibitory effects of certain codon pairs could be alleviated by the overexpression of a nonnative tRNA that precisely matched the target codon, while the overexpression of a native, wobble-decoding tRNA did not. Another study observed that certain codon pairs reduced translation elongation rates (122), an effect that could also be mitigated by overexpressing artificial, wobble-independent tRNAs. Thus, inhibitory codon pairs may reduce the rate at which the ribosome decodes wobble-containing codons, leading to ribosome stalling and collisions, relocation to stress granules, ribosome disassembly, and mRNA degradation via an RNase termed Cue2 (123) (termed N4BP2 in humans). While no link between codon- or codon pair-deoptimized viruses and ribosome stalling has yet been established, the disruption of coding biases may lead to virus attenuation via such a mechanism (Fig. 2).

Codon usage can also vary within individual genes and may be the result of selection against strong secondary structures near the translation initiation codon of mRNA molecules (124, 125). Some organisms appear to have evolved to repress codons that promote the formation of stable mRNA secondary structures since such structures can impact start codon recognition and translation initiation (126–128). Similarly, some viral genomes also exhibit apparent selection against strong secondary structures near initiation codons (129, 130). Another feature of 5′ regions of cellular genes is a “ramp” of poorly adapted codons whereby infrequently used codons that are putatively translated with low efficiency are enriched in the 5′ portion of open reading frames (ORFs), in contrast to 3′ regions (3). Indeed, ribosome density was found to be elevated in 5′ regions, implying slower transit. These biases may be a mechanism by which translation initiation is slowed and ribosomal “traffic jams” are avoided (131). The genomes of some viruses also show a preference for certain codons at the 5′ ends of genes (129, 132), but it is unclear whether selection pressures that impose these choices arise from the optimization of translation efficiency, GC content, or secondary structure. It is likely that current codon/codon pair deoptimization approaches could differentially impact translation depending on where in viral genomes or open reading frames they are applied.

Finally, another intragenic codon bias has been observed in eukaryotic ORFs, specifically the reuse of codons across an mRNA sequence, perhaps to increase the use of recycled tRNAs and facilitate translation (133). This pattern involves not only frequently used codons but also rare codons, and it is particularly apparent in rapidly induced genes, such as those involved in stress responses. It is possible that viruses might have evolved similar patterns to optimize the translation of their genes. Codon or codon pair deoptimization or optimization will likely disrupt these biases.

While mounting evidence in the field of RNA biology has described several protein complexes that detect and eliminate mRNA containing rare codons or codon pairs, it is unknown if the same pathways are involved in the recognition and degradation of recoded viruses. Approaches that combine specific genetic recoding of viruses and targeted gene perturbations, such as CRISPR-Cas9, will further our understanding of the role of these pathways in virus recoding-induced attenuation.

## CONCLUDING REMARKS

The increasing use of bioinformatic algorithms and access to large-scale DNA synthesis technology have powered the study of genetic coding biases in viruses. Modifications involving CpG and UpA dinucleotide enrichment led to the discovery of important antiviral mechanisms, such as ZAP-mediated and RNase L-mediated viral RNA degradation, which appear to be central mechanisms for the attenuation of recoded viruses. Codon and codon pair deoptimizations were among the first successful attempts to reduce virus replication by the introduction of synonymous mutations. Nevertheless, how much viral attenuation is due to codon pair-specific effects and how much is related to dinucleotide frequency modifications are still not completely understood. One way to address this question is to systematically genetically modify viral genomes by codon pair deoptimization and assess virus replication in cells where ZAP and RNase L are absent. Studying the replication and codon biases of organisms that lack orthologs of ZAP and RNase L may also illuminate attenuation mechanisms. Another important question that remains unaddressed is why, in some cases, codon optimization approaches can also lead to decreased replication of viruses. It is possible that codon optimization disrupts intragenic biases, as discussed above, but further investigation is required. Assessing whether manipulations impact not only translation but also mRNA stability will further our understanding of how genetic recoding leads to viral attenuation.

This relatively new field of synthetic virology has already yielded important discoveries. Further development of this area will expand the range of viral genome manipulations that lead to attenuation via novel mechanisms. Ultimately, this knowledge will impact the development of prophylactic interventions and improve the control of infectious diseases.
